# Prevalence and Clustering of Cardiovascular Disease Risk Factors among Tibetan Adults in China: A Population-Based Study

**DOI:** 10.1371/journal.pone.0129966

**Published:** 2015-06-05

**Authors:** Shaopeng Xu, Zepei Jiayong, Bin Li, Hong Zhu, Hong Chang, Wei Shi, Zhengxuan Gao, Xianjia Ning, Jinghua Wang

**Affiliations:** 1 Department of Cardiology, Tianjin Medical University General Hospital, Tianjin, China; 2 Department of Internal medicine, Changdu Region People’s Hospital, Tibet, China; 3 Department of Epidemiology, Tianjin Medical University, Tianjin, China; 4 Clinical laboratory, Changdu Region People’s Hospital, Tibet, China; 5 Laboratory of Epidemiology, Tianjin Neurological Institute & Department of Neurology, Tianjin Medical University General Hospital, Tianjin, China; University of Washington, UNITED STATES

## Abstract

**Objectives:**

The prevalence of cardiovascular disease risk factors has increased worldwide. However, the prevalence and clustering of cardiovascular disease risk factors among Tibetans is currently unknown. We aimed to explore the prevalence and clustering of cardiovascular disease risk factors among Tibetan adults in China.

**Methods:**

In 2011, 1659 Tibetan adults (aged ≥18 years) from Changdu, China were recruited to this cross-section study. The questionnaire, physical examinations and laboratory testing were completed and the prevalence of cardiovascular disease risk factors, including hypertension, diabetes, overweight/obesity, dyslipidemia, and current smoking, were counted. The association between the clustering of cardiovascular disease risk factors and demographic characteristics, and geographic altitude were assessed.

**Results:**

The age-standardized prevalence of hypertension, diabetes, overweight or obesity, dyslipidemia, and current smoking were 62.4%, 6.4%, 34.3%, 42.7%, and 6.1%, respectively, and these risk factors were associated with age, gender, education level, yearly family income, altitude, occupation, and butter tea consumption (P < 0.05). Overall, the age-adjusted prevalence of clustering of ≥1, ≥2, and ≥3 cardiovascular disease risk factors were 79.4%, 47.1%, and 20.9%, respectively. There appeared higher clustering of ≥2 and ≥3 cardiovascular disease risk factors among Tibetans with higher education level and family income yearly, and those living at an altitude < 3500 m and in a township.

**Conclusions:**

The prevalence of cardiovascular disease risk factors, especially hypertension, was high in Tibetans. Moreover, there was an increased clustering of cardiovascular disease risk factors among those with higher socioeconomic status, lamas and those living at an altitude < 3500 m. These findings suggest that without the immediate implementation of an efficient policy to control these risk factors, cardiovascular disease will eventually become a major disease burden among Tibetans.

## Introduction

Cardiovascular disease (CVD) is a leading cause of death in both developed and developing countries worldwide. The economic burden of coronary heart disease and stroke in 2009 were estimated to be $234 billion in the United States alone [1]. Although the prevalence of CVD risk factors has decreased in some developed countries, the prevalence has increased in developing countries [2–4].

Statistics from 2010 indicate that CVD is the leading cause of death in China; CVD accounted for nearly 42% of all deaths in 2010. In China, the economic burden of CVD is estimated to be $550 billion from 2005 to 2015[5]. The epidemiologic transition of CVD coincides with the increased prevalence of CVD risk factors, resulting from rapid economic development and lifestyle changes. Several studies have indicated that the prevalence of CVD risk factors have increased in China over past decades [6–8]. However, most studies of CVD risk factors have been performed in urban Han populations [9–11], the epidemiology of CVD in minority ethnic populations is lacking.

Recently, several studies on the prevalence of CVD and risk factors in Tibetans have indicated that the burden of CVD and relative risk factors has increased in this population over past decades [12–15]. However, these studies were conducted in the population residing at the same altitude, and excluded lamas. Thus, the prevalence and clustering of CVD risk factors among Tibetans residing at different altitudes, including lamas, are currently unknown.

For this study, we performed a population-based survey in a representative population of Changdu, China to evaluate the prevalence of CVD risk factors among Tibetans, including lamas and Tibetans living at different altitudes. We aimed to assess the epidemiology of CVD risk factors in a low-income, low-education Tibetan population.

## Methods

### Study population

The study population was recruited from the Changdu region in the Tibet Autonomous Region, China from September 2010 to June 2011. There are 11 counties, including142 townships and 11 central temples in the Changdu region with altitudes ranging from 3200 m to 4500 m. More than 95% of residents are Tibetan. A four-stage randomly stratified cluster sampling method was used to select a representative sample of the Tibetan population in China. First, all 11 counties in the Changdu region were stratified into three groups according to altitude: <3500 m, 3500–4000 m, and >4000 m. Second, one county was selected from each altitude group, which included three counties. Third, four townships from each selected county were selected, for a total of 12 townships. Fourth, three villages or neighborhoods from each selected township were selected, resulting in a total of 36 villages or neighborhoods. Moreover, one central temple was selected in each county; all qualified lamas were recruited in the present study. Finally, all residents aged ≥ 18 years from the selected 31 villages, 5 neighborhoods, and 3 central temples were recruited in this survey.

After excluding 135 residents with the confirmed history of CVD, a total of 650 households, including 2175 persons aged ≥ 18 years without a history of CVD (including stroke, angina, and myocardial infarction), in 31 villages, 5 neighborhoods, and 3 central temples met the selection criteria. A total of 1960 residents completed the survey and examination, response rate of 90.1%. Of these, a total of 1659 Tibetans were included in the present study, after excluding 134 residents without complete demographic data and 167 residents of other ethnic.

The ethics committee of Changdu Region People’s Hospital, Tibet approved the study, and written informed consent was obtained from all participants during recruitment.

### Data collection

The surveys were conducted in the community health station through face-to-face interviews, and physical examinations by trained research staff guided by epidemiology professionals. A few participants took the survey at home. A standardized questionnaire was administered in this survey. Detailed information included demographical information, including gender, age group (18–34 years, 35–44 years, 45–54 years, 55–64 years, ≥ 65 years), occupation (officer, workers, farmers, herdsman, lama, others), and education levels (0 years, 1–9 years, ≥ 10 years); socioeconomic status, including yearly family income (< 800 USD/year, 800–1599 USD/year, ≥ 1600 USD/year); geographical characteristics, including residence ((township, rural/ pastoral area, temple), and altitude (< 3500 m, ≥ 3500 m); life style factors, including cigarette smoking(no, yes) and butter tea consumption (no, yes); and self-reported previous medical history, medication use. The physical examinations included measurement of blood pressure (BP); body height and weight; and circumferences of the waist, hip, and abdomen. Additionally, all participants took an oral glucose tolerance test (OGTT), fasting glucose, total cholesterol (TC), triglycerides (TG), high-density lipoprotein cholesterol (HDL-C), and low-density lipoprotein cholesterol (LDL-C).

### Blood pressure measurement and hypertension definition

Subjects were advised to avoid smoking cigarettes, drinking alcohol, taking tea or coffee, or engaging in physical activity for at least 30 min before BP measurements. BP measurements were performed in a quiet room by senior physicians, using a calibrated mercury sphygmomanometer (Jiangsu Yuyue Medical Equipment co., Ltd), by the standard method of the American Hypertension Association [16]. Subjects were asked to remain resting in a sitting position for 5 min before testing. BP was recorded as the mean of two measurements taken 5 min apart after resting in the supine position, with cuff size adjusted to arm circumference. The cuff was placed on the arm at the level of the heart. If the difference between the two readings was over 5 mmHg, an additional two readings were obtained after resting for an additional 20 min. Systolic BP and diastolic BP were defined according to Korotkoff sounds I and V.

Hypertension was defined as an average systolic BP ≥ 140 mmHg and/or an average diastolic BP ≥ 90 mmHg and/or self-reported current treatment for hypertension with antihypertensive medication [17].

### Body weight and height measurements and obesity definition

Body weight and height were measured to the nearest tenth of a kilogram/meter by standard protocol and techniques, with light clothing and no shoes. Body mass index was calculated as weight (in kilograms) divided by the square of height (in meters). According to standard criteria in Chinese adults, overweight and obesity were defined as body mass indices of 24.0–27.9 kg/m^2^ and ≥28.0 kg/m^2^, respectively [18].

### Laboratory testing

All participants were examined serum lipids (TC, TG, HDL-C, and LDL-C) and plasma glucose levels, which were measured enzymatically in the Clinical laboratory of Changdu Region People’s Hospital, Tibet, which was the certificated laboratory for clinical examination, with reagents from Guangzhou Kefang Company, China using HITACHI 7180 Chemistry Analyzer (HITACHI Company, Japan)[19]. Dyslipidemia was defined as self-reported current treatment with cholesterol-lowering medication or having 1 or more of the following levels: TC ≥ 5.2 mmol/L, TG ≥ 1.7 mmol/L, HDL-C < 1.04 mmol/L, or LDL-C ≥3.4 mmol/L [20]. Plasma glucose was measured using a modified hexokinase enzymatic method. Individuals without a previous history of diabetes were administered an OGTT with 75 g glucose. Diabetes was defined as having a fasting plasma glucose level ≥ 7.0 mmol/L, OGTT ≥ 11.11 mmol/L, a self-reported history of diabetes, or current treatment with antidiabetes medication [21].

### Statistical analysis

The data were presented as means (standard difference) for continuous variables, and as percentages (95% confidence interval) for categorical variables. Categorical variables were analyzed using the Chi-squared test. The prevalence rates of CVD risk factors, which included hypertension, diabetes mellitus, overweight/obesity, dyslipidemia, and smoking, were standardized using the population according to the China sixth national population census, with the population divided into 9 age–groups: 18–34 years, 35–39 years, 40–44 years, 45–49 years, 50–54 years, 55–59 years, 60–64 years, 65–74 years, and ≥75 years [22]. The prevalence of each CVD risk factor, and CVD risk factor clustering (with 0, ≥1, ≥2, and ≥3 CVD risk factors) were described by gender, age group (18–34 years, 35–44 years, 45–54 years, 55–64 years, ≥ 65 years), education levels (0 years, 1–9 years, ≥ 10 years), yearly family income (< 800 USD/year, 800–1599 USD/year, ≥ 1600 USD/year), altitude (< 3500 m, ≥ 3500 m), residence (township, rural/ pastoral area, temple), and butter tea consumption(no, yes). The odd ratio (OR) for age-adjusted prevalence with ≥1, ≥2, and ≥3 CVD risk factors were estimated using Poisson regression. Statistical significance was defined as P < 0.05 two tailed. SPSS version 15.0 for Windows (SPSS Inc., Chicago, IL, USA) was used for the analyses.

## Results

### Descriptive characteristics of participants

The descriptive characteristics of all participants in this study are presented in Table 1. In this cross-sectional study, there were a total of 1659 subjects, including 822 (49.5%) men and 837 (50.5%) women; one-third of participants were aged 18–34 years; 59.5% of participants were farmers/herdsmen, and 54.7% lived at an altitude of 3200–3500 m. The education levels were lower both in men and in women, with 60% illiteracy overall. Among all participants, 56.9% had a yearly family income of <800 USD.

**Table 1:** The demographic characteristics among Tibetans aged 18 years and over in China.

Characteristics	Men	Women	Total
Number, n(%)	822(49.5)	837(50.5)	1659(100.0)
Age, years, means(SD)	41.5(15.0)	46.5(15.1)	44.0(15.2)
Age group, n(%):			
18–34 yrs	299(36.4)	194(23.2)	493(29.7)
35–44 yrs	194(23.6)	193(23.1)	387(23.3)
45–54 yrs	161(19.6)	201(24.0)	362(21.8)
55–64 yrs	105(12.8)	138(16.4)	243(14.7)
≥65 yrs	63(7.7)	111(13.3)	174(10.5)
Residence, n(%):			
Township	144(17.5)	251(30.0)	395(23.8)
Rural/ Pastoral area	353(43.0)	583(69.6)	936(56.4)
Temple	325(39.5)	3(0.4)	328(19.8)
Altitude, meter, n(%):			
< 3500	444(54.0)	463(55.3)	907(54.7)
3500–4000	266(32.4)	334(39.9)	600(36.2)
>4000	112(13.6)	40(4.8)	152(9.1)
Education, n(%):			
0 yrs	394(47.9)	607(72.6)	1001(60.3)
1–9 yrs	376(45.7)	145(17.3)	521(31.4)
≥10 yrs	52(6.3)	85(10.2)	137(8.3)
Occupation, n(%)			
Officer	56(6.8)	93(11.1)	149(9.0)
Workers	26(3.2)	15(1.8)	41(2.5)
Farmers	334(40.6)	582(69.5)	916(55.2)
Herdsman	31(3.8)	40(4.8)	71(4.3)
Shaman	337(41.0)	7(0.8)	344(20.7)
Others	38(4.6)	100(12.0)	138(8.3)
Family income yearly, n(%):			
< 800 USD	508(61.8)	436(52.1)	944(56.9)
800–1599 USD	145(17.6)	162(19.4)	307(18.5)
≥1600 USD	169(20.6)	239(28.5)	408(24.6)

### Age-standardized prevalence of primary CVD risk factors

Table 2 presents the age-standardized prevalence of the primary CVD risk factors, including hypertension, diabetes, overweight or obesity, dyslipidemia, and current smoking. The corresponding prevalence were 62.4%, 6.4%, 34.3%, 42.7%, and 6.1%, respectively. With the exception of hypertension, the prevalence of these CVD risk factors was greater in men than in women. There were significant increased trends with age in the prevalence of all of these risk factors; except for hypertension, risk factor prevalence showed increased trends with education levels; and except for hypertension and overweight/obesity, risk factor prevalence showed increased trends with yearly family income (P < 0.05). The prevalence of hypertension, diabetes, overweight/obesity, and current smoking were highest among Tibetans living at an altitude < 3500 m. Concurrently, the prevalence of diabetes and current smoking were higher in those living in a township; moreover, the prevalence of overweight/obesity was greater in lamas than in farmers/herdsmen (P < 0.05).

**Table 2:** The age adjusted prevalence of CVD risk factors by demographic characteristics among Tibetan adults. ^a^

Category	Hypertension	Diabetes mellitus	Overweight/Obesity	Dyslipidemia	Smoking
Total:	62.4(64.6)	6.4(6.8)	34.3(35.6)	42.7(43.8)	6.1(6.1)
Age group:					
18–34 yrs	34.9^b^	1.9^b^	18.5^b^	30.8^b^	7.1^b^
35–44 yrs	63.6	4.8	42.9	42.1	5.7
45–54 yrs	79.0	8.0	45.3	50.6	8.3
55–64 yrs	87.2	13.3	38.2	49.4	3.7
≥ 65 yrs	89.7	13.3	44.1	53.6	2.9
Education level:					
0 yrs	63.8(67.9)	5.4(6.0)^b^	28.9(30.6)^b^	39.2(40.8)^b^	3.9(3.6)^b^
1–9 yrs	60.3(59.7)	9.2(8.1)	43.5(43.5)	50.0(49.0)	8.4(8.5)
≥ 10 yrs	63.4(59.1)	8.8(7.4)	42.7(41.2)	51.4(45.9)	15.6(15.3)
Family income yearly:					
< 800 USD	61.9(61.7)	4.1(4.1)^b^	34.3(34.7)	38.8(39.1)^b^	4.1(4.2)^b^
800–1599 USD	67.0(71.7)	5.4(6.3)	38.4(41.3)	43.3(45.4)	8.4(7.8)
≥1600 USD	60.7(66.2)	11.3(13.0)	29.8(33.4)	49.9(52.6)	9.6(9.1)
Altitude:					
<3500 m	65.0(68.7)^b^	7.3(8.4)^b^	40.1(42.6)^b^	43.8(46.3)	8.3(8.4)^b^
≥3500 m	59.8(59.7)	4.9(5.0)	26.4(27.1)	40.4(40.9)	3.3(3.3)
Residence:					
Township	63.8(70.1)	9.8(12.5)^b^	44.6(50.4)^b^	47.2(52.0)	11.8(11.9)^b^
Rural/ Pastoral area	62.0(67.2)	5.3(5.7)	23.1(25.5)	36.6(38.7)	6.7(5.8)
Temple	63.6(50.6)	4.2(2.6)	59.5(46.9)	49.2(47.9)	0
Butter tea:					
No	62.4(65.2)	6.0(6.1)	44.8(45.3)	62.2(63.1)^b^	8.5(8.7)
Yes	62.3(64.6)	6.4(6.8)	33.7(35.2)	41.9(42.9)	6.0(6.0)

^a^ data was presented in adjusted prevalence (rude rate) except for age group.

^b^ indicated P<0.05 for chi-square testing or trend chi-square testing by group.

### Age-standardized prevalence of primary CVD risk factors by gender

The prevalence of CVD risk factors increased with age both in men and in women, but differed by demographic and socioeconomic groups. In the higher education level group, there were higher prevalence of overweight/obesity, dyslipidemia, and current smoking in men, but higher prevalence of diabetes, dyslipidemia, and current smoking in women. In the higher family income group, there were higher prevalence of all these risk factors except for overweight/obesity in men, but higher prevalence of diabetes only in women. Among those living at lower altitude, there were higher prevalence of hypertension, overweight/obesity, and current smoking in men, and overweight/obesity, dyslipidemia, and current smoking in women. Moreover, the prevalence of hypertension, diabetes, dyslipidemia, and current smoking were higher among those living in townships, and among men, the prevalence of overweight/obesity was higher in lamas; but only diabetes and overweight/obesity in women. There was a higher prevalence of dyslipidemia in men and current smoking in women among those who did not consume butter tea (Table 3).

**Table 3:** The age adjusted prevalence of CVD risk factors by demographic characteristics among Tibetan by gender. ^a^

	Hypertension		Diabetes mellitus		Overweight/Obesity		Dyslipidemia		Smoking	
	Men	Women	Men	Women	Men	Women	Men	Women	Men	Women
Overall:	65.3(64.1)	59.7(65.1)	7.9(7.7)	5.1(5.9)^b^	41.3(41.1)	27.1(30.1)^b^	51.1(51.1)	33.0(36.7)^b^	11.1(11.3)	1.0(1.0)^b^ ^b^ ^b^
Age group:										
18–34 yrs	36.8^c^	32.0^c^	2.5^c^	1.1^c^	22.8^c^	11.9^c^	40.8^c^	15.6^c^	11.0^c^	1.0^c^
35–44 yrs	66.5	60.6	5.3	4.3	52.9	32.8	52.9	31.0	10.3	1.0
45–54 yrs	83.9	75.1	11.7	5.1	54.7	37.7	61.0	42.4	18.0	0.5
55–64 yrs	88.6	86.2	14.1	12.7	41.8	35.6	44.4	53.0	6.7	1.5
≥ 65 yrs	95.2	86.5	19.0	10.2	55.6	37.2	56.9	51.9	6.3	0.9
Education level:										
0 yrs	66.8(67.3)	61.9(68.4)	6.6(6.9)	4.5(5.4)^c^	35.5(36.1)^c^	24.1(27.1)	47.9(48.3)^c^	32.0(36.0)^c^	8.5(8.4)^c^	0.4(0.5)^c^
1–9 yrs	61.7(58.5)	56.4(62.8)	9.1(7.9)	11.9(8.6)	46.8(45.3)	35.2(38.9)	52.7(51.7)	38.4(42.0)	10.6(10.6)	3.4(2.8)
≥ 10 yrs	77.7(80.8)	53.5(45.9)	9.8(11.5)	11.6(4.8)	48.7(49.2)	36.1(36.5)	66.0(67.3)	45.7(32.5)	35.7(38.5)	1.5(1.2)
Family income yearly:										
< 800 USD	62.7(57.3)^c^	61.7(66.7)	5.1(4.9)^c^	2.8(3.1)^c^	42.8(40.9)^c^	25.6(27.4)	45.8(46.3)^c^	39.1(27.6)	7.3(7.1)^c^	0.9(0.9)
800–1599 USD	72.6(75.2)	61.3(68.5)	5.1(5.6)	5.4(6.9)	45.1(47.1)	32.6(36.1)	51.6(52.4)	35.1(39.0)	15.9(15.2)	1.6(1.2)
≥1600 USD	69.8(75.1)	55.2(59.8)	14.2(17.2)	8.8(10.0)	34.3(36.9)	27.6(30.9)	65.8(63.3)	41.0(45.0)	24.2(20.7)	0.8(0.8)
Altitude:										
<3500 m	70.8(72.5)^c^	59.1(65.0)	8.5(9.1)	6.4(7.7)	48.1(49.4)^c^	32.5(36.0)^c^	51.1(51.8)	36.3(41.1)^c^	14.8(15.3)^c^	1.8(1.7)^c^
≥3500 m	59.4(54.2)	60.4(65.2)	6.6(6.2)	3.4(3.7)	31.9(31.4)	20.4(22.8)	48.9(50.3)	28.7(31.4)	6.1(6.6)	0
Residence:										
Township	74.9(81.3)^c^	58.1(63.7)	12.7(16.7)^c^	8.4(10.0)^c^	50.0(57.5)^c^	41.5(46.3)^c^	64.6(68.1)^c^	37.2(42.7)	31.8(29.2)^c^	1.7(2.0)
Rural/Pastoral area	65.2(70.0)	60.2(65.5)	8.1(8.5)	3.7(4.1)	26.7(29.3)	19.6(23.1)	45.2(46.2)	31.3(34.2)	16.8(14.4)	0.6(0.5)
Temple	63.3(50.2)	24.4(100)	4.3(2.6)	0	59.7(46.9)	8.7(50.0)	49.9(46.9)	0	0	0
Butter tea:										
No	76.4(76.7)	43.5(56.4)^c^	10.0(10.3)	2.4(2.7)	53.8(56.0)	33.2(38.5)	78.7(85.7)^c^	36.9(47.2)	10.9(10.0)	6.7(7.7)^c^
Yes	64.8(63.6)	60.5(65.5)	7.8(7.6)	5.2(6.0)	40.9(49.9)	32.7(36.2)	49.8(49.9)	32.7(36.2)	11.2(11.4)	0.7(0.3)

^a^ data was presented in adjusted prevalence (rude rate) except for age group.

^b^ indicated P<0.05 compared between men and women for chi-square testing.

^c^ indicated P<0.05 for chi-square testing or trend chi-square testing by group.

### Age-standardized prevalence in clustering of primary CVD risk factors

Overall, 20.3% of Tibetan adults had no CVD risk factors; this included 14.4% of men and 27.7% of women, and this difference was statistically significant. The prevalence rates of ≥1, ≥2, and ≥3 primary CVD risk factors were 79.4%, 47.1%, and 20.9% overall, 82.1%, 54.9%, and 28.9% in men, 72.5%, 38.8%, and 13.7% in women, respectively. The proportion of those without risk factors was higher in women than in men (27.7% vs 14.4%, P<0.001), however, there were higher proportions of those with ≥2 and ≥3 primary CVD risk factors in men than in women (P< 0.001). With increasing age, there was a trend toward increased prevalence rates of ≥1, ≥2, and ≥3 primary CVD risk factors both in men and in women (P < 0.001). In men, there was an obvious increased trend toward clustering of ≥2 and ≥3 primary CVD risk factors with increasing education level, yearly family income, and altitude, in addition to butter tea consumption; in women, there was an obvious increased trend toward clustering of ≥ 2 and ≥ 3 primary CVD risk factors with increasing education level and altitude, in addition to residing in township. The prevalence of those with ≥3 primary CVD risk factors was greater among those with higher yearly family income in women, and among those living in a township in men (Tables 4 and 5).

**Table 4:** The age adjusted prevalence with different numbers of CVD risk factors among Tibetan adults. ^a^

Category	0 Risk factors	≥1 Risk factors	≥2 Risk factors	≥3 Risk factors
Total:	20.3(18.8)	79.4(81.2)	47.1(49.3)	20.9(22.0)
Age group:				
18–34 yrs	39.0^b^	61.0^b^	25.5^b^	8.7^b^
35–44 yrs	16.8	83.2	51.2	22.4
45–54 yrs	8.7	91.3	62.3	28.7
55–64 yrs	5.3	94.8	62.7	28.9
≥ 65 yrs	6.3	93.8	66.3	35.6
Education level:				
0 yrs	20.5(17.9)	79.5(82.1)	43.7(46.7)^b^	15.9(17.8)^b^
1–9 yrs	18.6(18.6)	81.4(81.4)	52.6(53.1)	29.0(28.1)
≥ 10 yrs	20.5(25.4)	79.5(74.6)	59.6(53.7)	32.9(29.9)
Family income yearly:				
< 800 USD	20.5(20.7)	79.5(79.3)	44.5(45.0)	15.3(17.8)^b^
800–1599 USD	21.0(18.1)	79.0(81.9)	51.1(55.3)	26.3(29.0)
≥1600 USD	19.5(15.1)	80.5(84.9)	50.0(54.3)	22.9(26.1)
Altitude:				
<3500 m	19.2(16.5)	80.8(83.5)	53.6(57.5)^b^	26.2(28.6)^b^
≥3500 m	21.6(21.4)	78.4(78.6)	39.3(40.0)	14.3(14.6)
Residence:				
Township	20.9(15.6)	79.1(84.4)	55.8(63.5)	30.4(35.7)
Rural/ Pastoral area	22.1(18.6)	77.9(81.4)	40.3(43.6)	14.1(15.7)
Temple	17.2(23.3)	82.8(76.7)	61.6(47.9)	31.0(23.3)
Butter tea:				
No	20.3(16.7)	79.7(83.3)	60.0(63.3)^b^	30.5(31.7)^b^
Yes	20.3(18.9)	79.7(81.1)	46.7(48.7)	20.6(21.7)

^a^ data was presented in adjusted prevalence (rude rate) except for age group.

^b^ indicated P<0.05 for chi-square testing or trend chi-square testing by group.

**Table 5:** The age-adjust prevalence with different numbers of CVD risk factors among Tibetan men and women. ^a^

	0 Risk factors		≥1 Risk factors		≥2 Risk factors		≥3 Risk factors	
	Men	Women	Men	Women	Men	Women	Men	Women
Overall:	14.4(14.7)	27.7(22.7)^b^	82.1(85.3)	72.5(77.3)^b^	54.9(54.9)	38.8(43.9)^b^	28.9(28.3)	13.7(15.9)^b^
Age group:								
18–34 yrs	28.9^c^	54.1^c^	71.1^c^	46.0^c^	33.2^c^	14.1^c^	12.6^c^	2.7^c^
35–44 yrs	11.6	22.0	88.4	78.0	60.8	41.4	32.8	11.8
45–54 yrs	3.3	13.0	96.7	87.1	73.0	53.9	43.4	17.1
55–64 yrs	4.1	6.1	95.9	93.9	66.0	60.3	27.8	29.8
≥ 65 yrs	5.2	6.9	94.8	93.1	72.4	62.7	50.0	27.5
Education level:								
0 yrs	13.7(13.5)	26.4(20.8)	86.3(86.5)	73.6(79.2)	51.7(52.6)^c^	36.8(43.0)^c^	23.4(23.7)^c^	12.0(14.0)^c^
1–9 yrs	15.8(17.1)	30.1(22.6)	84.2(82.9)	71.0(77.4)	55.9(54.1)	44.4(50.4)	31.4(29.6)	23.2(24.1)
≥ 10 yrs	7.8(7.8)	27.0(36.1)	88.5(92.2)	73.0(63.9)	73.9(76.5)	50.4(40.0)	53.2(52.9)	25.6(15.7)
Family income yearly:								
< 800 USD	16.0(18.8)^c^	27.4(22.9)	84.0(81.2)^c^	72.6(77.2)	51.4(49.0)^c^	35.6(40.3)	24.7(23.1)^c^	10.1(11.8)^c^
800–1599 USD	12.0(13.0)	28.3(22.6)	85.8(87.0)	71.7(77.4)	58.4(60.9)	44.6(50.3)	34.6(36.2)	18.7(22.6)
≥1600 USD	5.8(4.8)	27.5(22.6)	94.2(95.2)	72.5(77.5)	65.8(66.1)	41.0(46.0)	33.9(36.3)	16.2(18.7)
Altitude:								
<3500 m	12.7(15.4)	26.4(21.2)	83.6(84.6)	73.6(78.8)	63.3(65.3)^c^	42.1(50.1)^c^	35.6(36.6)^c^	17.8(21.0)^c^
≥3500 m	16.3(18.4)	29.2(24.5)	80.0(81.6)	70.8(75.6)	44.5(43.4)	35.2(36.5)	19.8(19.2)	8.5(9.9)
Residence:								
Township	8.2(5.0)^c^	27.1(21.8)	91.8(95.0)^c^	72.9(78.2)	72.7(78.7)	47.9(54.7)^c^	46.8(51.8)^c^	22.0(26.3)^c^
Rural/ Pastoral area	12.0(10.9)	27.6(23.2)	87.5(89.1)	72.0(76.8)	48.6(51.1)	35.0(39.1)	20.9(22.8)	10.0(11.4)
Temple	17.3(23.4)	0	82.7(76.6)	17.3(100)	61.9(47.9)	18.7(50.0)	31.2(23.4)	0
Butter tea:								
No	0^c^	40.0(27.8)	100(100)^c^	60.0(72.2)	77.5(79.2)^c^	40.7(52.8)	40.2(41.7)	19.6(25.0)
Yes	14.8(15.2)	27.2(22.5)	85.2(84.8)	72.8(77.5)	54.2(54.1)	38.6(43.5)	28.5(27.9)	12.7(15.5)

^a^ data was presented in adjusted prevalence (rude rate) except for age group

^b^ indicated P<0.05 compared between men and women for chi-square testing.

^c^ indicated P<0.05 for chi-square testing or trend chi-square testing by group.

## Discussion

This is the first study to report the prevalence and clustering of main CVD risk factors by demographic, geographic, and life style characteristics among Tibetans in China. We assessed the prevalence of CVD risk factors by demographical characteristics, including gender, age groups, education levels, yearly family income; and by geographical features, including occupation, altitude, and butter tea consumption among Tibetans living at altitudes 3200–4500 m in Changdu, China.

Tibetans are an ethnic group with a unique lifestyle characterized by a special local diet of tsampa, butter tea, beef, and mutton, which contain high salts and cholesterols; moreover, the majority of Tibetans reside at high altitudes.

This cross-sectional study indicated that the prevalence of hypertension, diabetes, overweight/obesity, dyslipidemia, and current smoking among Tibetan adults were 62.4%, 6.4%, 34.3%, 42.7%, and 6.1%, respectively. The prevalence of these CVD risk factors increased significantly with age, with higher rates in men than in women, with the exception of hypertension. The prevalence rates of diabetes, overweight/obesity, dyslipidemia, and current smoking increased with education level; the prevalence rates of diabetes, dyslipidemia, and current smoking increased with yearly family income. Moreover, there appeared to be a higher prevalence of hypertension, diabetes, overweight/obesity, and current smoking in Tibetans living at altitudes < 3500 m than those living at altitudes ≥ 3500 m; there were higher prevalence rates of diabetes and current smoking in those living in townships, and higher prevalence rates of overweight/obesity in lamas, and higher prevalence rates of dyslipidemia in those who did not consumed butter tea.

A previous study indicated that the age-standardized prevalence rates of dyslipidemia, hypertension, diabetes, current smoking, and overweight in Chinese adults aged 35–74 years were 53.6%, 26.1%, 5.2%, 34.4%, and 28.2%, respectively [23]. Recently, several studies reported a higher prevalence of CVD risk factors in Han adults. In Beijing adults aged 35–74 years in 2007, the prevalence rates of hypertension, diabetes, overweight, dyslipidemia, and current smoking were 36.9%, 6.5%, 36.2%, 35.4%, and 36.3%, respectively [24]. Another cross-sectional survey in rural residents aged 40 years and over in Beijing found that the prevalence rates of hypertension, diabetes, and overweight were 47.2%, 7.7%, and 53.3% in men, respectively, and 44.8%, 8.2%, and 60.7% in women, respectively, from 2008 to 2010 [25]. In the present study, the prevalence rate of hypertension was higher than other minority groups in China (52.6% among Kazaks, 54.6% among Uyghurs) [26,27].

The high prevalence of hypertension in Tibetans in this study may in part be due to unique physiologic changes in residents living at high altitudes, which involve respiratory, cardiovascular, and hematologic adaptations to long-term hypoxemic conditions. In addition, increased hemoglobin, hematocrit, and leukocytes are associated with the atherosclerotic process and hypertension [28,29]. Moreover, the diets of Tibetans may have contributed in part to the high prevalence of hypertension observed in this study.

The association of socioeconomic factors with CVD risk factors has been indicated in previous studies. The prevalence of diabetes, obesity, smoking, and dyslipidemia decreased with increasing socioeconomic status [30–34]. However, the prevalence of diabetes, obesity, dyslipidemia, and current smoking in the present study showed an upward trend with socioeconomic status, consistent with a previous report [35]. The delayed economic development in Tibet may explain partly the association between high education levels and yearly family income with the high prevalence of CVD risk factors. Moreover, the socioeconomic classes in this study were stratified according to the local economic development; however, Tibetans are all in a lower socioeconomic class.

A great number of studies have demonstrated that CVD incidence and all-cause mortality increased markedly in the presence of risk factor clustering [36,37]. In National Health and Nutrition Examination Survey Epidemiologic Follow-up Study, the age-, race-, sex-, and education-adjusted relative risks of coronary heart disease in adults with 1, 2, 3, or 4 or 5 risk factors (high blood pressure, high cholesterol, diabetes, overweight, and current smoking) were 1.6, 2.2, 3.1, and 5.0 during 21 years of follow-up, respectively, compared to those with no risk factors [38].

The studies determined that 80.5%, 45.9%, and 17.2% of Chinese adults had ≥1, ≥2, and ≥3 CVD risk factors, respectively. The clustering of risk factors was approximately equal for men and women and increased with age [23]. In the present study, the prevalence rates of clustering of ≥1 and ≥2 CVD risk factors were79.4% and 47.1%, respectively, but the prevalence of clustering of ≥3 CVD risk factors was greater than that reported in a prior study (20.9% vs. 17.2%) [23]. There are fewer Tibetans smoking because of Tibetan Buddhism restrictions, thus, the actual risk of CVD risk factor clustering in Tibetans was greater than that in Han adults.

In the present study, we found that the clustering of CVD risk factors was associated with education level, family income, altitude, occupation, and butter tea consumption. There were greater risks of clustered CVD risk factors in Tibetans with a high education level and family income, those living at altitudes < 3500 m or in a township, and in those who never consumed butter tea.

These results may be explained by the delayed economic development in Tibet and the protected role of tea. However, at the initial stage of rapid economic development in Tibet, the quality of life improved only among Tibetans with higher socioeconomic status. This delayed economic development may drive the greater risk of CVD in the wealthy.

Our study had several limitations. First, the population size was relatively small because of the limited Tibetan population in the region; however, the four-stage randomly stratified cluster sampling method was used to make the study population representative. Second, all participants were from altitudes of ≥3200 m, and we did not include a population at a lower altitude for purposes of comparison. However, we conducted subgroup analyses stratified by altitude (<3500 m and ≥3500 m) for analyses. Third, our study demonstrated that the prevalence of CVD risk factors was associated with yearly family income, but as Tibet is an undeveloped region, the level of economic development in this population was lower overall. Thus, although the level of income in Tibetans is relatively low, yearly family income was stratified into three groups (< 800 USD, 800–1600 USD, and >1600 USD). Finally, because of religious beliefs, Tibetans do not consume alcohol; therefore, we were unable to calculate the prevalence of alcohol consumption as a CVD risk factor.

## Conclusion

This is the first cross-sectional study to evaluate the prevalence and clustering of CVD risk factors among Tibetan adults in China. The prevalence of primary CVD risk factors, including hypertension, diabetes, overweight/obesity, dyslipidemia, and current smoking, were high in Tibetans. Moreover, there was a greater proportion of clustering of ≥3 CVD risk factors in Tibetans. Poor socioeconomic status and residence situations contribute to Tibetans having a higher CVD risk. Our findings suggest that the incidence of CVD in Tibetans may continue to increase over future decades; without the immediate implementation of an efficient policy to control risk factors, CVD will eventually become a major disease burden among Tibetans.
